# A high-quality chromosome-level genome assembly of the bivalve mollusk *Mactra veneriformis*

**DOI:** 10.1093/g3journal/jkac229

**Published:** 2022-09-27

**Authors:** Yongxin Sun, Xiangfeng Liu, Xi Xie, Yongan Bai, Shuo Wang, Weiming Teng, Dacheng Li, Hualin Li, Zuoan Yu, Ming Zhang, Zunchun Zhou, Xu Liu, Hongtao Nie, Shaojun Du, Xiaodong Li, Qi Li, Qingzhi Wang

**Affiliations:** Dalian Key Laboratory of Genetic Resources for Marine Shellﬁsh, Liaoning Ocean and Fisheries Science Research Institute, Dalian 116023, China; Dalian Key Laboratory of Genetic Resources for Marine Shellﬁsh, Liaoning Ocean and Fisheries Science Research Institute, Dalian 116023, China; Dalian Key Laboratory of Genetic Resources for Marine Shellﬁsh, Liaoning Ocean and Fisheries Science Research Institute, Dalian 116023, China; Panjin Guanghe Crab Industry Co., LTD, Panjin 124299, China; Dalian Key Laboratory of Genetic Resources for Marine Shellﬁsh, Liaoning Ocean and Fisheries Science Research Institute, Dalian 116023, China; Dalian Key Laboratory of Genetic Resources for Marine Shellﬁsh, Liaoning Ocean and Fisheries Science Research Institute, Dalian 116023, China; Dalian Key Laboratory of Genetic Resources for Marine Shellﬁsh, Liaoning Ocean and Fisheries Science Research Institute, Dalian 116023, China; Dalian Key Laboratory of Genetic Resources for Marine Shellﬁsh, Liaoning Ocean and Fisheries Science Research Institute, Dalian 116023, China; Dalian Key Laboratory of Genetic Resources for Marine Shellﬁsh, Liaoning Ocean and Fisheries Science Research Institute, Dalian 116023, China; Dalian Key Laboratory of Genetic Resources for Marine Shellﬁsh, Liaoning Ocean and Fisheries Science Research Institute, Dalian 116023, China; Dalian Key Laboratory of Genetic Resources for Marine Shellﬁsh, Liaoning Ocean and Fisheries Science Research Institute, Dalian 116023, China; Panjin Guanghe Crab Industry Co., LTD, Panjin 124299, China; College of Fisheries and Life Science, Dalian Ocean University, Dalian 116023, China; Department of Biochemistry and Molecular Biology, Institute of Marine and Environmental Technology, University of Maryland School of Medicine, Baltimore, MD 21202, USA; Key Laboratory of Zoonosis, Aquaculture Department, College of Animal Science and Veterinary Medicine, Shenyang Agricultural University, Shenyang 110866, China; Key Laboratory of Mariculture, Ministry of Education, Ocean University of China, Qingdao 266003, China; Dalian Key Laboratory of Genetic Resources for Marine Shellﬁsh, Liaoning Ocean and Fisheries Science Research Institute, Dalian 116023, China

**Keywords:** genome assembly, chromosome, synteny, bivalve, *Mactra veneriformis*

## Abstract

*Mactra veneriformis* (Bivalvia: Mactridae) is a bivalve mollusk of major economic importance in China. Decreased natural yields of *M. veneriformis* have led to an urgent need for genomic resources. To address this problem and the currently limited knowledge of molecular evolution in this genus, we here report a high-quality chromosome-level genome assembly of *M. veneriformis*. Our approach yielded a 939.32 Mb assembled genome with an N50 contig length of 7,977.84 kb. Hi-C scaffolding of the genome resulted in assembly of 19 pseudochromosomes. Repetitive elements made up ∼51.79% of the genome assembly. A total of 29,315 protein-coding genes (PCGs) were predicted in *M. veneriformis*. Construction of a genome-level phylogenetic tree demonstrated that *M. veneriformis* and *Ruditapes philippinarum* diverged around 231 million years ago (MYA). Inter-species comparisons revealed that 493 gene families have undergone expansion and 449 have undergone contraction in the *M. veneriformis* genome. Chromosome-based macrosynteny analysis revealed a high degree of synteny between the 19 chromosomes of *M. veneriformis* and those of *Patinopecten yessoensis*. These results suggested that *M. veneriformis* has a similar karyotype to that of *P. yessoensis*, and that a highly conserved 19-chromosome karyotype was formed in the early differentiation stages of bivalves. In summary, the genomic resources generated in this work serve as a valuable reference for investigating the molecular mechanisms underlying biological functions in *M. veneriformis* and will facilitate future genetic improvement and disease treatment in this economically important species. Furthermore, the assembled genome greatly improves our understanding of early genomic evolution of the Bivalvia.

## Introduction

Bivalves comprise approximately 20,000 species that are distributed throughout aquatic habitats (Beckman 2012). The clam *Mactra veneriformis* is a type of infaunal suspension-feeding bivalve that is ubiquitous and abundant in tidal flats, subtidal areas, and shallow seas along the coast of China, Korea, and Japan (Guan *et al.* 2021) and is of major economic importance in China (Fang *et al.* 2010). In addition to its high value as a food, the shell and fresh meat of *M. veneriformis* are also used in traditional folk medicine (Yuhai 1996). *M. veneriformis* yield has recently experienced a sharp decline due to over-fishing (Hou *et al.* 2006) and potentially to altered immune function as a result of stress associated with acute temperature changes (Yu *et al.* 2009). *M. veneriformis* germplasm resource protection has therefore become an urgent task. The complete mitochondrial genome of *M. veneriformis* has been assembled and was shown to have noncoding regions, a unique characteristic compared with the mitochondrial genomes of other Veneroida species (Meng *et al.* 2013). Genes potentially involved in shell color patterns and shell shape were identified in recent studies (Yan *et al.* 2019). The molecular mechanisms underlying the tolerance of benthic organisms to diverse biotic and abiotic stressors, such as parasites, ocean acidification, and hypoxia, have received much attention in recent years (Kodama *et al.* 2018; Lim *et al.* 2006; Zhao *et al.* 2018).

In the last decade, the number of genomic resources available for clams has risen exponentially; genomes have been assembled for bivalves such as *Patinopecten yessoensis* (Wang *et al.* 2017), *Scapharca (Anadara) broughtonii* (Bai *et al.* 2019), *Ruditapes philippinarum* (Yan *et al.* 2019), *Crassostrea gigas* (Qi *et al.* 2021), *Tegillarca granosa* (Bao *et al.* 2021), and *Scapharca kagoshimensis* (Teng *et al.* 2022). Those achievements have greatly promoted efforts to understand genetic diversity and fine variety breeding, and to protect germplasm resources. However, despite its commercial importance, the genome of the clam *M. veneriformis* has not yet been sequenced.

In this study, we address this important knowledge gap by reporting a chromosome-level genome sequence of *M. veneriformis* assembled using a combination of Illumina short read sequencing, PacBio single-molecule sequencing, and Hi-C sequencing. The genome assembly has a high level of completeness and will serve as an excellent resource for future genomic, biological, and ecological studies on this species. In addition, the assembled genome will facilitate future breeding for improved phenotypes such as high yield, rapid growth, and robust disease resistance.

## Materials and methods

### Samples and DNA sequencing

*Mactra veneriformis* samples were collected from Dalian City, Liaoning Province, China. Genomic DNA was extracted using the TIANamp DNA Kit following the manufacturer’s instructions (Tiangen, Beijing, China). DNA was then sheared using a sonication device to enable the construction of short-insert paired-end (PE) libraries. The short-insert (500 bp) libraries were constructed following the instructions provided with the Illumina Nextera DNA Library Prep Kit. All libraries were sequenced on an Illumina X-TEN platform (San Diego, CA, USA). After the Illumina sequencing adaptors were removed, low-quality bases at the ends of the raw reads were trimmed. Reads were then scanned using a 4-bp sliding window for further trimming using an average quality per base threshold of 15. The resulting clean data were used in downstream analyses. For long-read sequencing, a PacBio SMRTbell library (20 kb) for PacBio Sequel was constructed using the SMRTbell Express Template Prep Kit 2.0 (PacBio, Menlo Park, CA, USA). Fragments smaller than 7 kb were filtered out using BluePippin (Sage Science, MA, USA). The library was bound to polymerase using the Sequel II Binding Kit 2.0 (PacBio) and loaded onto a PacBio Sequel II sequencing platform using HiFi Bundle (v2) sequencing reagent and 8M SMRT cells. *M. veneriformis* muscles fixed in 1% (v/v) formaldehyde were used for in situ Hi-C library construction. Nucleus extraction, permeabilization, chromatin digestion, proximity-ligation treatments, and subsequent DNA manipulation were performed as described previously (Lieberman-Aiden *et al.* 2009). Mbol was used for chromatin digestion. The Hi-C libraries were sequenced on the Illumina X-TEN platform with 2 × 150 bp cycle configuration. To facilitate the prediction of PCGs, RNA was extracted using the TRNzol Universal Reagent. RNA-Seq libraries were prepared using the NEBNext Ultra RNA Library Prep Kit (New England Biolabs, Ipswich, MA, USA) following the manufacturer’s instructions, then sequenced on an Illumina X-TEN platform. Quality control of the resulting raw reads was performed using Trimmomatic (Bolger *et al.* 2014).

### Genome size, heterozygosity estimation, and genome assembly

The genome size and heterozygosity of *M. veneriformis* were determined using quality-filtered reads and GenomeScope (Vurture *et al.* 2017) at *k *=* *17. Accordingly, we used Jellyfish (v2.2.10) (Marçais and Kingsford 2011) to obtain *k*-mer (*k* = 17) depth distribution, and roughly estimated the genome size by dividing the total number of *k*-mers by their respective coverage.

We integrated the assembled PacBio HiFi reads and PE Hi-C reads using Hifiasm (Cheng *et al.* 2021) with default parameters. The PE Illumina short reads were then aligned to the corrected Hifiasm contigs with BWA-MEM v0.7.17 (Li and Durbin 2010). The assembly was polished with Pilon v1.2 (Walker *et al.* 2014) to correct errors in the contigs. Hi-C data were independently analyzed using the HiC-Pro pipeline (Servant *et al.* 2015) with default parameters and LIGATION_SITE set to “GATC.” The 3D-DNA pipeline (Dudchenko *et al.* 2017) was used to assign the order and orientation of each group, and distribution of links among chromosomes was visualized with a heatmap using HiCPlotter (Akdemir and Chin 2015). We further evaluated the completeness of the genome assembly and gene set using Benchmarking Universal Single-Copy Orthologs (BUSCO) (Simão *et al.* 2015).

### Genome annotation

We employed RepeatModeler to ensure the integrity of the assembled genes and to identify repeat families. RepeatMasker (http://www.repeatmasker.org; accessed 2022 September 8) was used to perform de novo identification and masking of the repeat sequences in the assembly. To identify protein-coding regions, we obtained annotations via de novo prediction, homology-based searches, and transcriptome-assisted methods. We used the de novo gene prediction packages Augustus v3.2.3 (Stanke *et al.* 2008) and GeneMark v4.32 (Borodovsky *et al.* 2003) to perform the ab initio coding gene prediction with repeat-masked genome sequences. For homology-based prediction, we used homologous reference protein sequences of *Dreissena polymorpha*, *Crassostrea gigas*, *Mizuhopecten yessoensis*, and *Daphnia pulex* from the National Center for Biotechnology Information (NCBI) genome database and aligned them to the *M. veneriformis* genome assembly using BLASTP (Altschul *et al.* 1990) with the parameters “-evalue 1e^−5^.” Subsequently, the homology-based gene prediction program Exonerate v2.2.0 (Slater and Birney 2005) was used to build gene structures. For transcriptome-based predictions, we mapped the RNA-Seq data against the assembly using Tophat as described in the software documentation (Trapnell *et al.* 2009). Cufflinks (Trapnell *et al.* 2012) was then used on the transcripts to perform gene model analysis using the parameter “-multi-read-correct.” We integrated the data generated from the 3 prediction methods using EvidenceModeler (EVM) (Haas *et al.* 2008) and obtained a nonredundant (nr) gene set. Functional annotations were assigned in this gene set through alignment to the NCBI nr, SwissProt, and Eukaryotic Orthologous Genes (KOG) databases using BLASTP with an *e*-value of 1e^−5^. Protein domains were mapped against the InterPro and Pfam databases using InterProScan (Jones *et al.* 2014) and HMMER (Finn *et al.* 2011). The putative pathways to which genes belonged were determined using the Kyoto Encyclopedia of Genes and Genomes (KEGG) database. The Gene Ontology (GO) terms for each gene were extracted from the corresponding InterProscan or Pfam results.

### Phylogenetic analysis and divergence time estimation

We identified the types of orthologous relationships between the protein sequences from 20 selected species using Orthofinder (Emms and Kelly 2019). Protein sequences were also searched against the NCBI nr database using BLASTP and the BLOSUM62 matrix with a threshold *e*-value of 1e^−5^. The sequences were then clustered using Markov clustering with an inflation value of 1.5. Orthologous proteins were aligned using MUSCLE (Edgar 2004), then concatenated into a unique multiple sequence alignment using a custom Perl script. A neighbor-joining phylogenetic tree was reconstructed using MEGA5 software. To evaluate divergence time in the genome-based tree, phylogeny was calibrated in r8s (Sanderson 2003). Three calibration points were fixed in the molecular clock analysis. We estimated the molecular clock and the divergence times using a combined analysis with r8s and RAxML (Stamatakis 2014). The maximum-likelihood (ML) phylogenetic tree was constructed based on the General Time Reversible + Invariant + gamma sites (GTR + I + G) model of nucleotide substitution with 1,000 bootstrap replicates. The fossil-derived timescale and the evolutionary history of these species were obtained from TimeTree (Kumar *et al.* 2017).

### Expansion and contraction of gene families

To understand the evolutionary relationship between *M. veneriformis* and other bivalve species, taxonomic information for each of compared species has to be provided using a separate taxon label in phylogenetic tree (Fig. 3b). Gene gains and losses are the primary contributors to functional changes between species; we therefore sought to better understand the evolutionary dynamics of gene gains and losses in bivalves by determining the expansion and contraction of orthologous gene clusters among these 20 species. CAFE (De Bie *et al.* 2006) was used to perform this analysis using the topological gene tree. Random birth and death process models were used to identify gene gain and loss events on each branch of the phylogenetic tree. Hypergeometric tests were performed to determine whether specific GO functional categories were significantly overrepresented in *M. veneriformis* gene sets within the genome. Expanded and contracted gene families (in comparison to ancestors) were identified in different species and compared to those in *M. veneriformis* to understand gene family evolution in this species.

### Chromosomal macrosynteny analysis

*M. veneriformis* and *P. yessoensis* were separated in the early stages of bivalve evolution. *P. yessoensis* possessing a highly conserved 19-chromosome karyotype similar to that of bilaterian ancestors. To determine whether *M. veneriformis* also had the conservative chromosome collinearity relationship, macrosynteny analysis was performed on *M. veneriformis* and *P. yessoensis*. A comparison of gene synteny between *M. veneriformis* and *P. yessoensis* was conducted to visualize putative homologous gene pairs or marker pairs as inferred by sequence similarity. BLASTP was used in the gene-to-gene comparison with default *e*-values of 1e^−5^. A macrosynteny conservation index was calculated as measure of conservation (Simakov *et al.* 2013).

## Results and discussion

### Sequencing, genome size estimation, and genome assembly

**Fig. 1.**
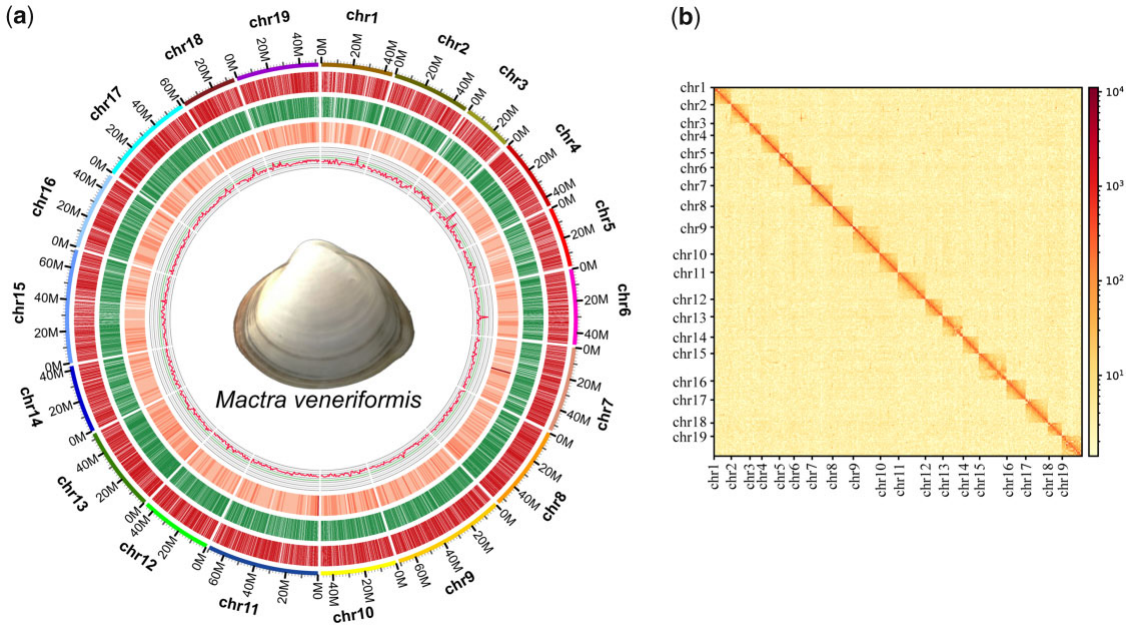
a) Global genome landscape of *Mactra veneriformis*. b) Hi-C interaction heat map for *M. veneriformis*. *Explanation notes below (a)*: From the outermost to the innermost circle: 19 chromosomes comprising the *M. veneriformis* genome at the Mb scale, showing genes on the forward strand in red and genes on the reverse strand in green; the calculated gene density per Mb; and GC content.

To obtain a high-quality chromosome-level genome assembly, we conducted deep genome sequencing of a single *M. veneriformis* individual. Raw reads were generated using 3 sequencing approaches, which yielded 28.3 Gb of cleaned Illumina short PE reads, 417.4 Gb of PacBio HiFi reads, and 81.3 Gb of Hi-C PE reads (Supplementary Table 1). Using all of the raw sequencing reads (representing ∼500× coverage of the estimated genome size) allowed us to obtain a high-quality chromosome-level genome assembly. PE sequencing library data and *k*-mer frequency analysis were then used to estimate the genome size of *M. veneriformis* as 865.63 Mb (Supplementary Fig. 1). Based on *k*-mer analysis, the genome scale heterozygosity of *M. veneriformis* was estimated to be 3.34%. Approximately 92.90% of the obtained sequences were successfully anchored to 19 pseudochromosomes, consistent with the haploid karyotypes of *T. granosa* (Bao *et al.* 2021) and *S. broughtonii* (Bai *et al.* 2019). The genome size of the final assembly for *M. veneriformis* was 979.32 Mb with an N50 contig length of 7.98 Mb (Fig. 1a). BUSCO results showed over 98.4% coverage of the metazoan single-copy ortholog gene set (Table 1 and Supplementary Table 3 and Supplementary Fig. 2). These results indicate that the assembled reads covered most of the coding regions, confirming the high quality and completeness of the genome assembly.

**Table 1.** Assembly statistics of *M. veneriformis* genome.

Assembly feature	Value
Length of total assembly (bp)	979,316,154
Largest scaffold length (bp)	26,817,445
Number of contigs	790
Contigs N50 (bp)	7,977,837
Numbers of N50	36
Contigs N90 (bp)	1,183,686
Numbers of N90	152
GC content (%)	33.26
N content (%)	0.00

### Genome annotation

**Fig. 2.**
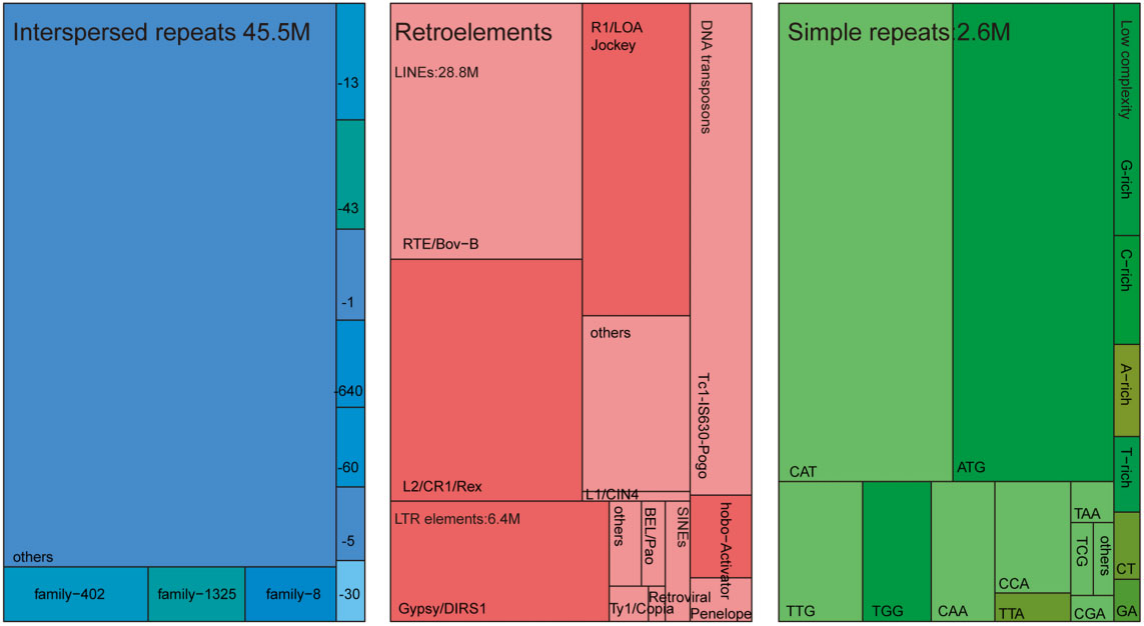
Treemap showing the categories of repetitive elements found in the *M. veneriformis* genome assembly using RepeatModeler coupled with RepeatMasker. *Explanation notes*: The left panel (blue) represents sequences masked based on species-specific repeat families. The middle panel (pink) shows repeats classified by RepeatModeler as long interspersed elements (LINEs), long terminal repeats (LTRs), and DNA transposons. The right panel (green) represents simple repeats and low complexity regions, with the most abundant dinucleotides and trinucleotides shown as individual categories.

Repetitive elements made up a large portion of the *M. veneriformis* genome, constituting ∼51.79% of the assembly; this included 0.06% satellites, 0.13% rolling-circles, 0.26% simple repeats, and 51.33% interspersed repeats. Among the latter, long interspersed elements (LINEs) and DNA transposable elements represented 2.94% and 1.27%, respectively. In contrast, long terminal repeat (LTR) elements represented only 0.65% of the assembly (Fig. 2 and Supplementary Table 2 and Supplementary Fig. 3). It was reported that tandem repeat elements occupied 7.9% of the pearl oyster (*Pinctada fucata*) genome (Takeuchi *et al.* 2012), but 36% of the pacific oyster *Crassostrea gigas* (Zhang *et al.* 2012). In this context, Murgarella *et al.* (2016) have revealed that repeat content varies in mollusks and is correlated with genome size.

We then employed transcriptome data and homolog-based approaches to predict gene models. The identified PCGs were integrated by EVM into a weighted and nr gene structure consensus model. A total of 29,315 PCGs were identified in *M. veneriformis* with an average coding sequence (CDS) length of 1526.9 bp (Table 2). PCG functional annotation was based on sequence similarity. Identified PCGs were subsequently aligned with those in several public databases (NCBI nr, GO [Supplementary Fig. 4], Swiss, KOG [Supplementary Fig. 5], and KEGG) using BLASTP. We mapped a total of 28,839 genes (98.38%) to at least 1 database, with 11,590 genes annotated in all 4 databases (Supplementary Fig. 6).

**Table 2.** Statistics of gene model feature for *M. veneriformis*.

Type	*M. veneriformis*
Genome size (bp)	979,316,154
Repeat sequence (bp)	507,219,442
Gene number	29,315
Gene density (gene_number/100 kb)	2.99
Gene average length (bp)	1,526.9
Exon number per gene	6.90
Intron number per gene	5.90
Exon average length (bp)	221.1
Intron average length (bp)	1800.5
Genome GC (%)	33.26
Exon GC (%)	36.89
Intergenic region average length (bp)	21,256.8

### Phylogenetic and divergence analyses

**Fig. 3.**
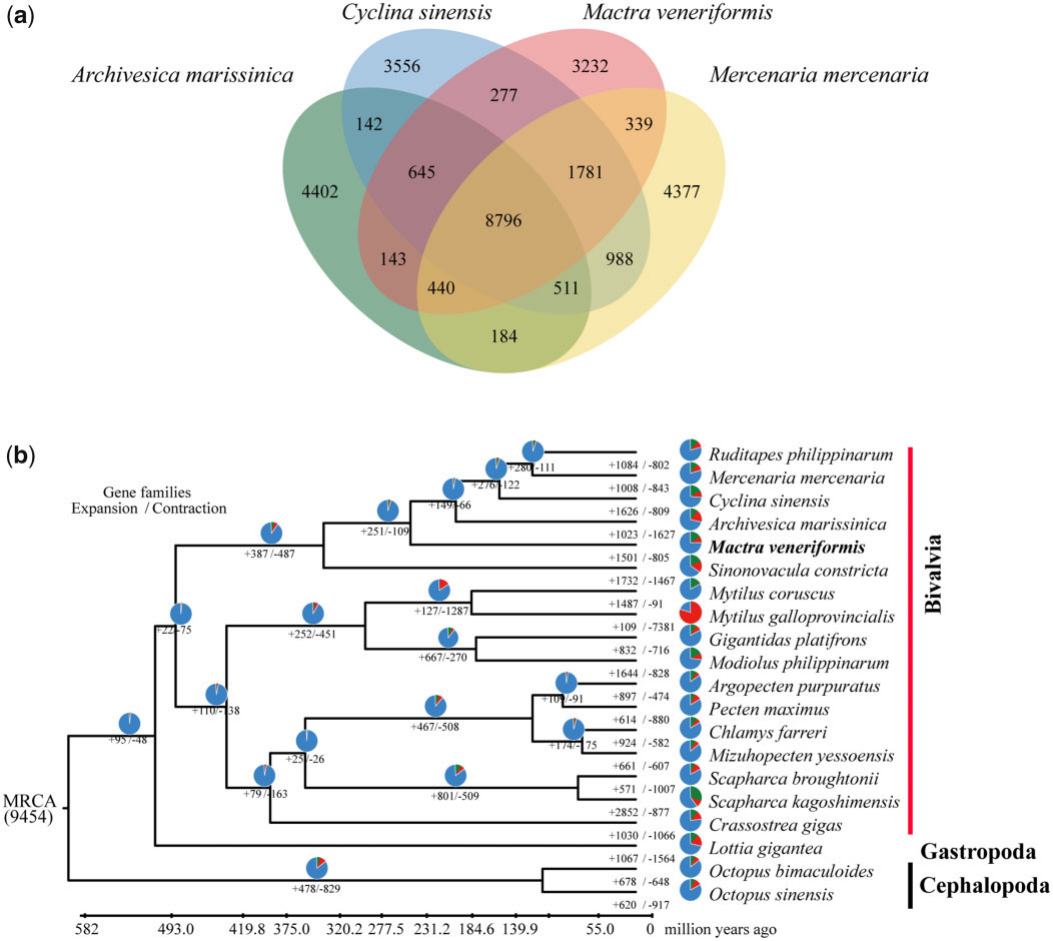
a) Four-way Venn diagram showing orthologous gene families shared between *Archivesica marissinica* (green), *Cyclina sinensis* (blue), *Mactra veneriformis* (pink), and *Mercenaria* (orange). b) Maximum-likelihood phylogenetic tree showing divergence points and the number of shared orthologs between *M. veneriformis* and 19 other mollusks. *Explanation notes below (b)*: The number of gene families undergoing expansion and contraction in each lineage are shown in red and green, respectively.

A phylogenetic tree was constructed to examine the evolutionary relationships between *M. veneriformis* and 19 other species. Among 20 mollusk species (17 bivalve, 1 gastropod, and 2 cephalopod species) analyzed, a total of 101,358 gene families were identified, 403 of which were present in all 20 species (Fig. 3a and Supplementary Table 4). Compared with the method of identifying gene families using annotated databases such as EggNOG and PhylomeDB, Orthfinder conducts BLAST all-versus-all searches across proteomes to infer groups of putatively orthologous genes, and can also find orthogroups and orthologs, infers rooted gene trees for all orthogroups and identifies all of the gene duplication events in those gene trees.

There were 65 single-copy genes found in all of the selected species, and these were used to build the phylogenetic tree. A ML phylogenetic tree was then constructed using RAxML (Fig. 3b). The results showed that bivalves were divided into 3 groups. The first group included *R. philippinarum*, *M. mercenaria*, *C. sinensis*, *A. marisinica*, and *M. veneriformis*. The second group included *S. constricta*, *M. coruscus*, *M. galloprovincialis*, *B. platifrons*, and *M. philippinarum*. The third group included *A. purpratus*, *P. maximus*, *C. farreri*, *M. yessoensis, S. broughtonii*, *S. kagoshimensis*, and *C. gigas.* Based on the phylogenomic analysis and the fossil record, we were able to date the divergence time between *M. veneriformis* and *R. philippinarum* to approximately 231 MYA.

### Expansion and contraction of gene families in the *Mactra veneriformis* genome

To compare different genomic traits between species, we performed a comparative genomic analysis of the 20 selected species using CAFE (Supplementary Table 5). We found that 1,501 and 805 gene families were significantly expanded and contracted in the *M. veneriformis* genome, respectively (*P < *0.05). Furthermore, the expanded and contracted gene families contained a total of 184 and 219 significantly enriched KEGG pathways, respectively (FDR < 0.05). The enrichment analyses suggested that the expanded gene families were involved in biological processes associated with pathogen responses and disease resistance (Supplementary Fig. 7).

### Chromosomal macrosynteny analysis

**Fig. 4.**
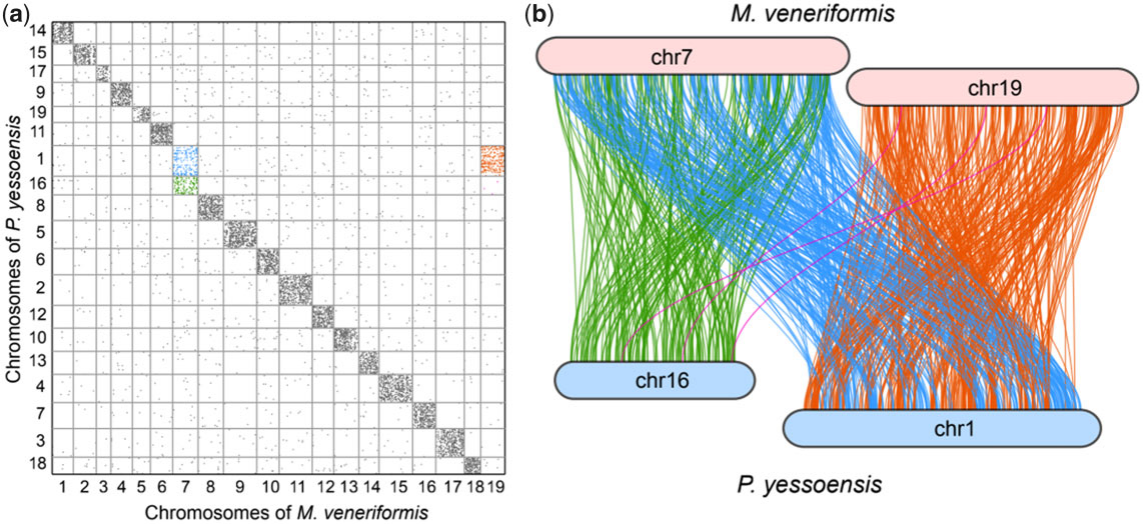
a) Syntenic dot plot illustration of orthologous genes between *Mactra veneriformis* and *Patinopecten yessoensis*. b) Collinearity between *P. yessoensis* chromosomes 01 and 16 and *M. veneriformis* chromosomes 07 and 19. *Explanation notes below (a)*: The horizontal axis represents chromosomes in *M. veneriformis* and the vertical axis represents chromosomes in *P. yessoensis*. *Explanation notes below (b)*: Orthologous gene pairs are connected by lines with the color representing the chromosome pair involved; orange corresponds to chromosome 19 of *M. veneriformis* and chromosome 01 of *P. yessoensis*; pink represents chromosome 19 of *M. veneriformis* and chromosome 16 of *P. yessoensis*; blue indicates chromosome 07 of *M. veneriformis* and chromosome 01 of *P. yessoensis*; green represents chromosome 07 of *M. veneriformis* and chromosome 16 of *P. yessoensis*.

Previous studies have shown that scallops and many other bivalves, such as *P. yessoensis* and *T. granosa*, have a highly conserved 19-chromosome karyotype (Thiriot-Quievreux 2002; Bao *et al.* 2021). In this study, *M. veneriformis* was also shown to have the 19-chromosome karyotype; however, the results of the phylogenetic analyses showed that *M. veneriformis* and *P. yessoensis* were in different groups of bivalves, meaning that the 2 species separated in the early stages of bivalve evolution. To determine whether *M. veneriformis* also had the conservative chromosome collinearity relationship, macrosynteny analysis was performed on *M. veneriformis* and *P. yessoensis*. The results showed high karyotype conservation between *M. veneriformis* and *P. yessoensis*, with the latter possessing a highly conserved 19-chromosome karyotype similar to that of bilaterian ancestors (Simakov *et al.* 2020). We identified chromosome fission or fusion events between the 19 chromosomes of *M. veneriformis* and those of *P. yessoensis*. Chromosome 01 of *P. yessoensis* corresponded to chromosomes 07 and 19 of *M. veneriformis*, whereas chromosome 07 of *M. veneriformis* corresponded to chromosomes 01 and 16 of *P. yessoensis* (Fig. 4a). To clarify the relationship between *P. yessoensis* chromosomes 01 and 16 and *M. veneriformis* chromosomes 07 and 19, we performed a collinearity analysis of those 4 chromosomes. The results showed that chromosome fission or fusion events may have been caused by a Robertsonian translocation (Fig. 4b). Previous studies by Bao *et al.* (2021) showed that *P. yessoensis* chromosome 01 also corresponded to 2 chromosomes of *T. granosa*, supporting the results of the present study. The results also suggest that Robinson chromosome translocation may have occurred in the *P. yessoensis* genome. The genomic collinearity analysis showed that there were few inter-chromosomal translocations between the genomes of *M. veneriformis* and *P. yessoensis*, whereas intra-chromosomal translocations occurred more frequently. This may be the explanation for the highly conserved 19-chromosome karyotype observed among bivalves.

### Conclusion

In this study, we assembled the first known high-quality chromosome-level genome for *M. veneriformis* using a combined strategy encompassing 3 distinct sequencing technologies: Illumina short reads, PacBio single-molecule sequencing, and Hi-C. We described features of the newly assembled genome and performed phylogenetic and gene family evolution analyses using 19 mollusk relatives. We performed chromosomal macrosynteny analysis and identified chromosomal fusion or fission events between the genomes of *M. veneriformis* and *P. yessoensis*, and determined that bivalves have a relatively low frequency of inter-chromosome translocation and a high frequency of intra-chromosome translocation. This newly assembled *M. veneriformis* genome constitutes an excellent resource for genomic, biological, and ecological studies of bivalve mollusks. High-quality genomic data for this species will contribute to development of molecular breeding techniques to generate bivalves with high yield, rapid growth, and disease resistant phenotypes with favorable economic traits.

## Supplementary Material

jkac229_Supplemental_Material_Legends

jkac229_Supplementary_Figure_S1

jkac229_Supplementary_Figure_S2

jkac229_Supplementary_Figure_S3

jkac229_Supplementary_Figure_S4

jkac229_Supplementary_Figure_S5

jkac229_Supplementary_Figure_S6

jkac229_Supplementary_Figure_S7

jkac229_Table_S1

jkac229_Table_S2

jkac229_Table_S3

jkac229_Table_S4

jkac229_Table_S5

## Data Availability

The *Mactra veneriformis* assembly is publicly available at GenBank with the accession number PRJNA836699. The associated raw sequencing data (both Illumina and PacBio HiFi) are available at the NCBI Sequence Read Archive (SRA) under the accession numbers SRR19160137, SRR19160138, and SRR19160139, respectively. Supplemental material is available at G3 online.
