# Supplementary material for: A high-quality chromosome-level genome assembly of the bivalve mollusk Mactra veneriformis
Source: G3 (Bethesda). 2022 Sep 27;12(11):jkac229. doi: 10.1093/g3journal/jkac229 (PMC9635629; doi:10.1093/g3journal/jkac229)
Supplement: jkac229_Supplemental_Material_Legends [file jkac229_supplemental_material_legends.docx]

**Supplemental Legends**

Figure S1 GenomeScope Profile.

Figure S2 BUSCO Assessment Results.

Figure S3 Interspersed Repeat Landscape.

Figure S4 Genes annotated in GO database.

Figure S5 KOG Function Classification

Figure S6 Genes annotated in all four databases.

Figure S7 Enriched pathway and GO description of expansion and contraction of gene families.

Table S1: Summary statistics for the clean sequencing data obtained from M. veneriformis via Illumina and PacBio sequencing.

Table S2: Summary statistics for the repeat elements found in the M. veneriformis genome assembly using both the RepeatModeler and RepeatMasker software.

Table S3: Estimation of core genes in the M. veneriformis genome assembly using the BUSCO software.

Table S4: Summary of the orthologous gene clusters analyzed in the 20 different species.

Table S5: Results of the gene family analysis implemented using the CAFE software.
