# Supplementary figures and images for: A high-quality chromosome-level genome assembly of the bivalve mollusk Mactra veneriformis
Source: G3 (Bethesda). 2022 Sep 27;12(11):jkac229. doi: 10.1093/g3journal/jkac229 (PMC9635629; doi:10.1093/g3journal/jkac229)

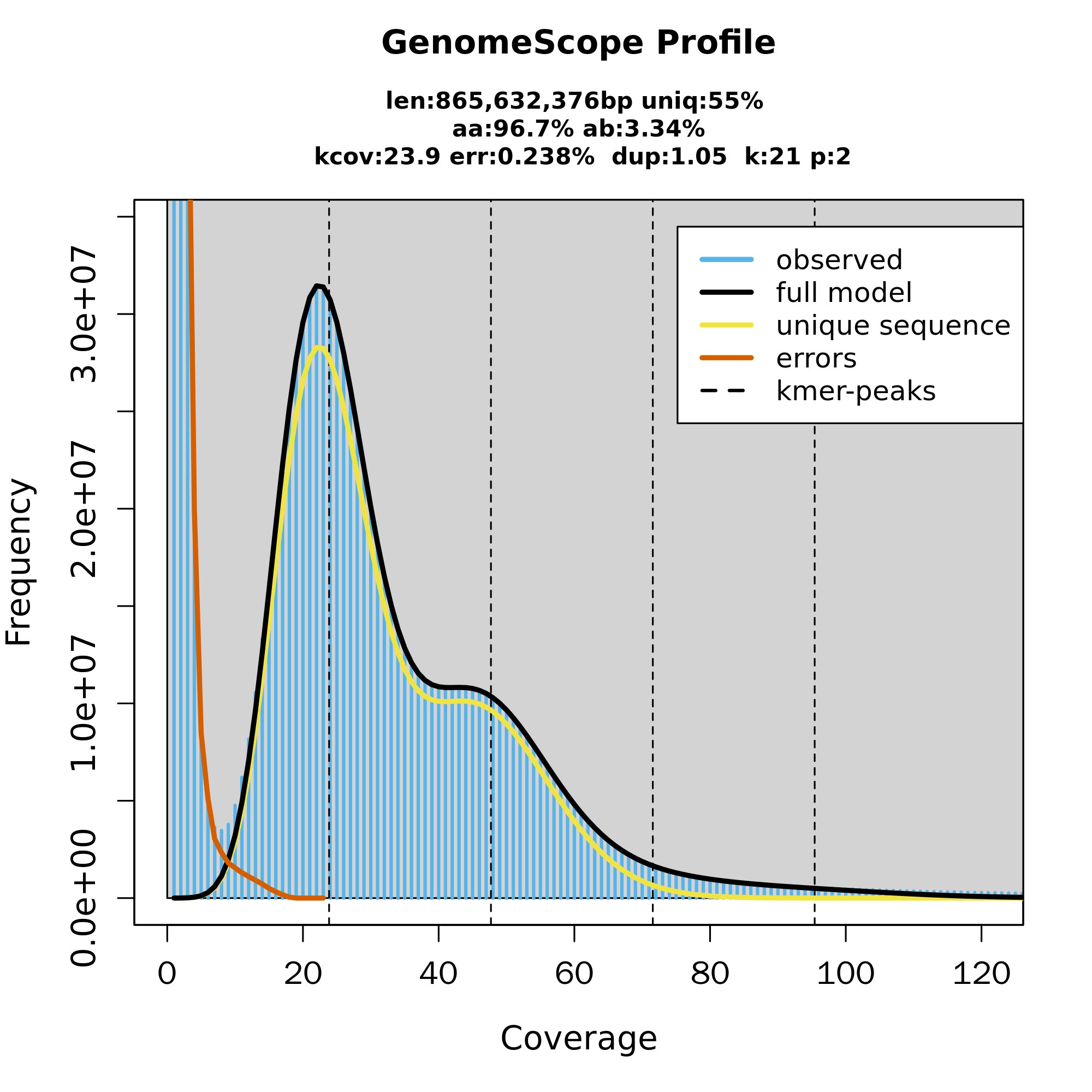

Supplement: jkac229_Supplementary_Figure_S1 [file jkac229_supplementary_figure_s1.jpeg]

# BUSCO Assessment Results

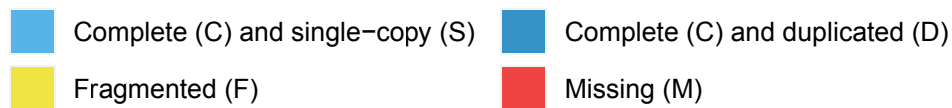

*Mactra sp.*

C:243 [S:213, D:30], F:6, M:6, n:255

0 20 40 60 80 100

%BUSCOs

Supplement: jkac229_Supplementary_Figure_S2 [file jkac229_supplementary_figure_s2.pdf]

# Interspersed Repeat Landscape

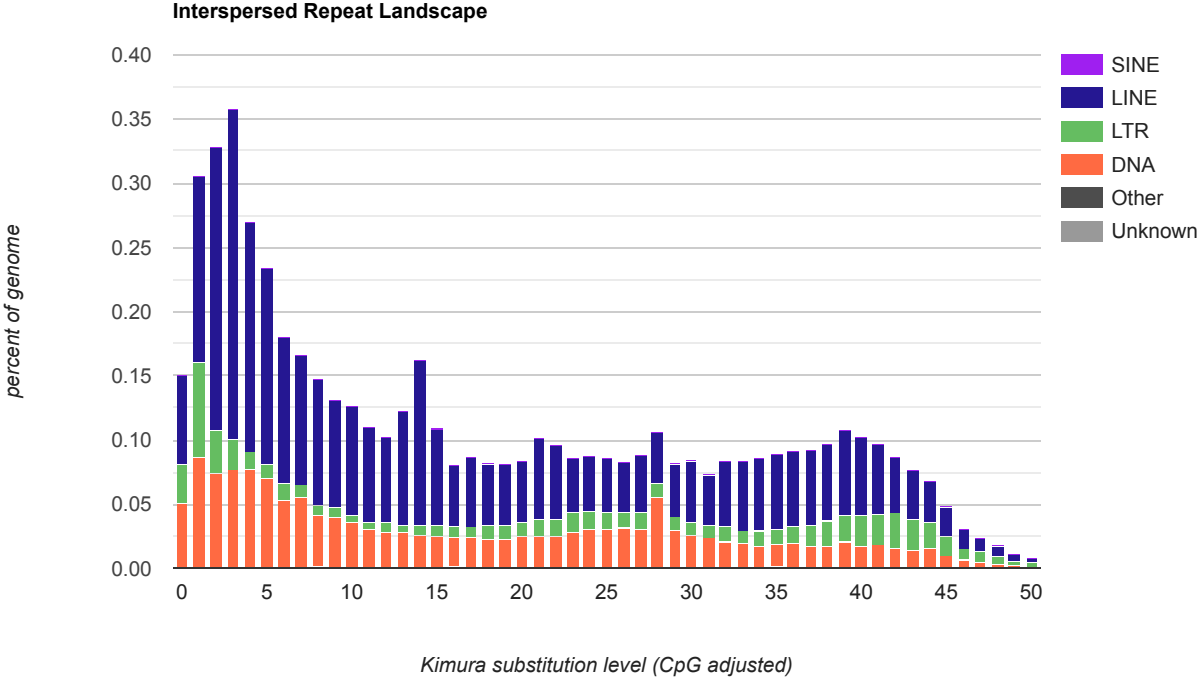

Supplement: jkac229_Supplementary_Figure_S3 [file jkac229_supplementary_figure_s3.pdf]

# WEGO Output

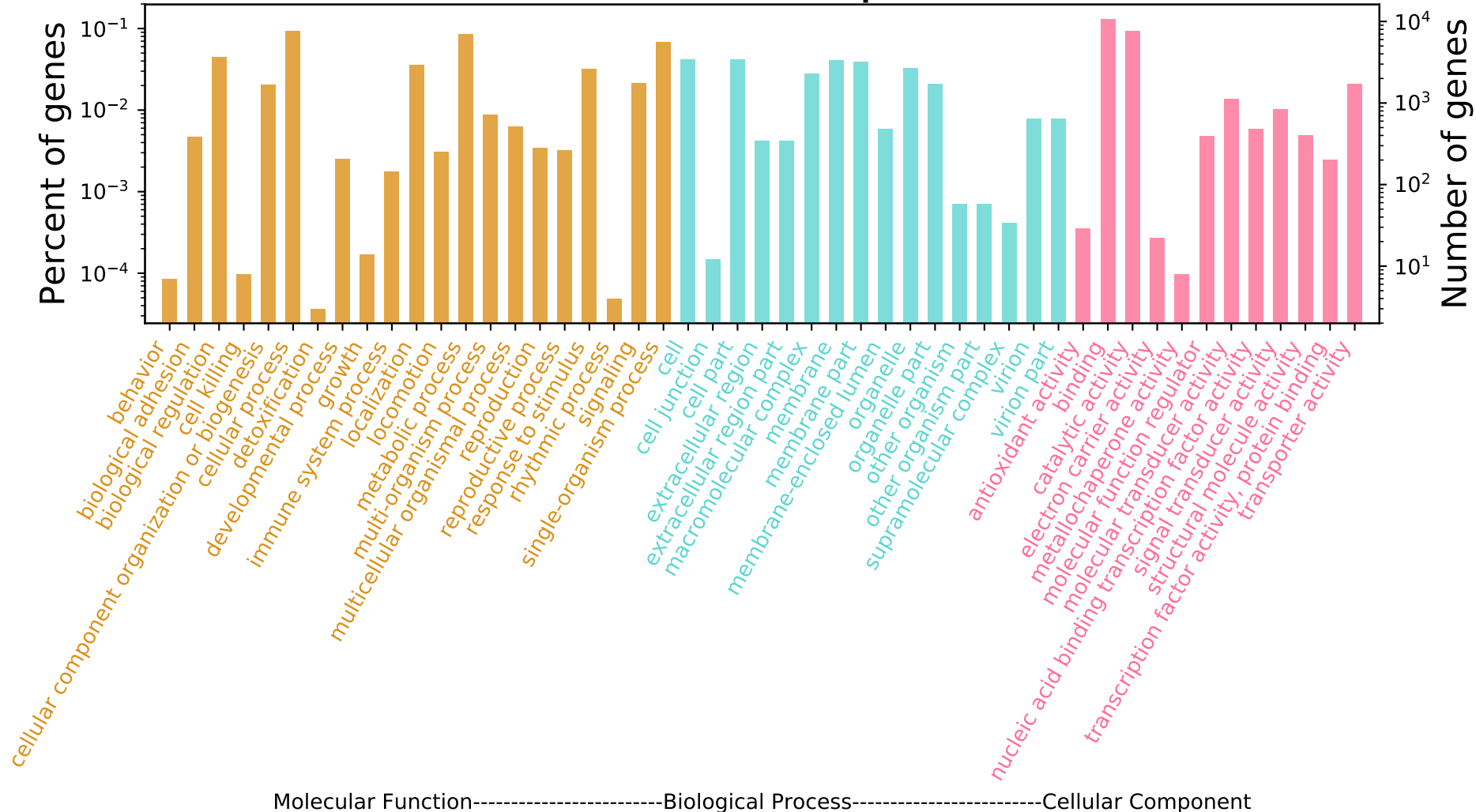

Supplement: jkac229_Supplementary_Figure_S4 [file jkac229_supplementary_figure_s4.pdf]

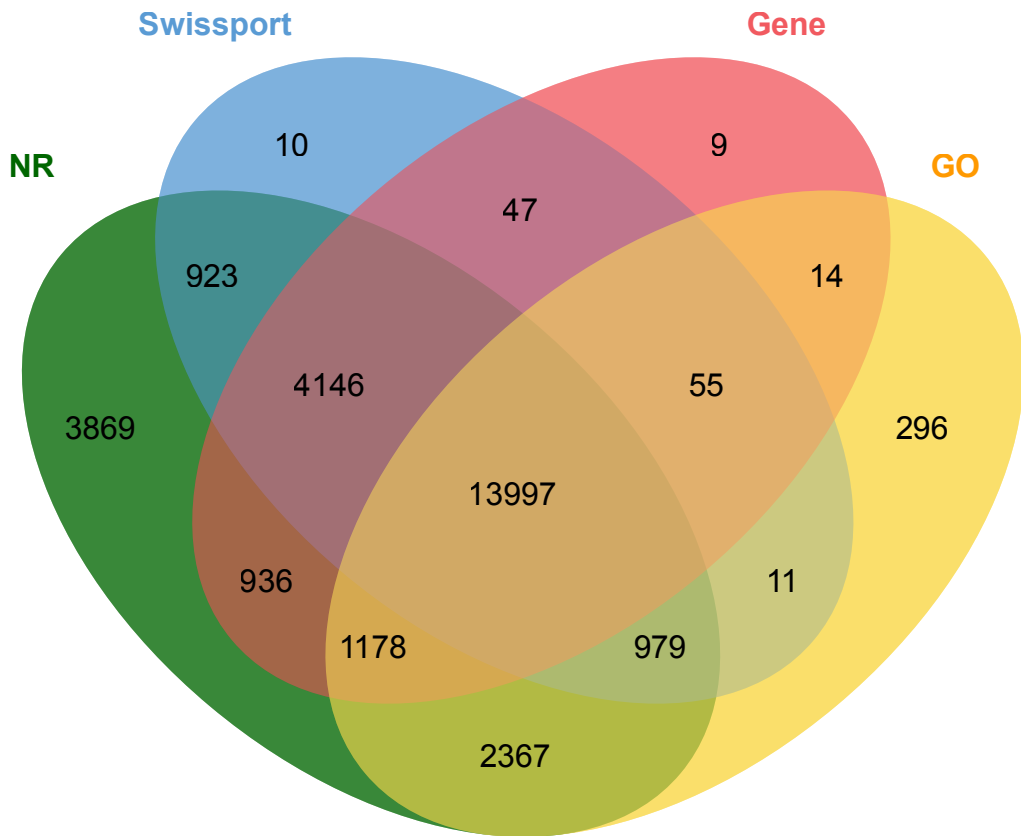

Supplement: jkac229_Supplementary_Figure_S6 [file jkac229_supplementary_figure_s6.pdf]

A

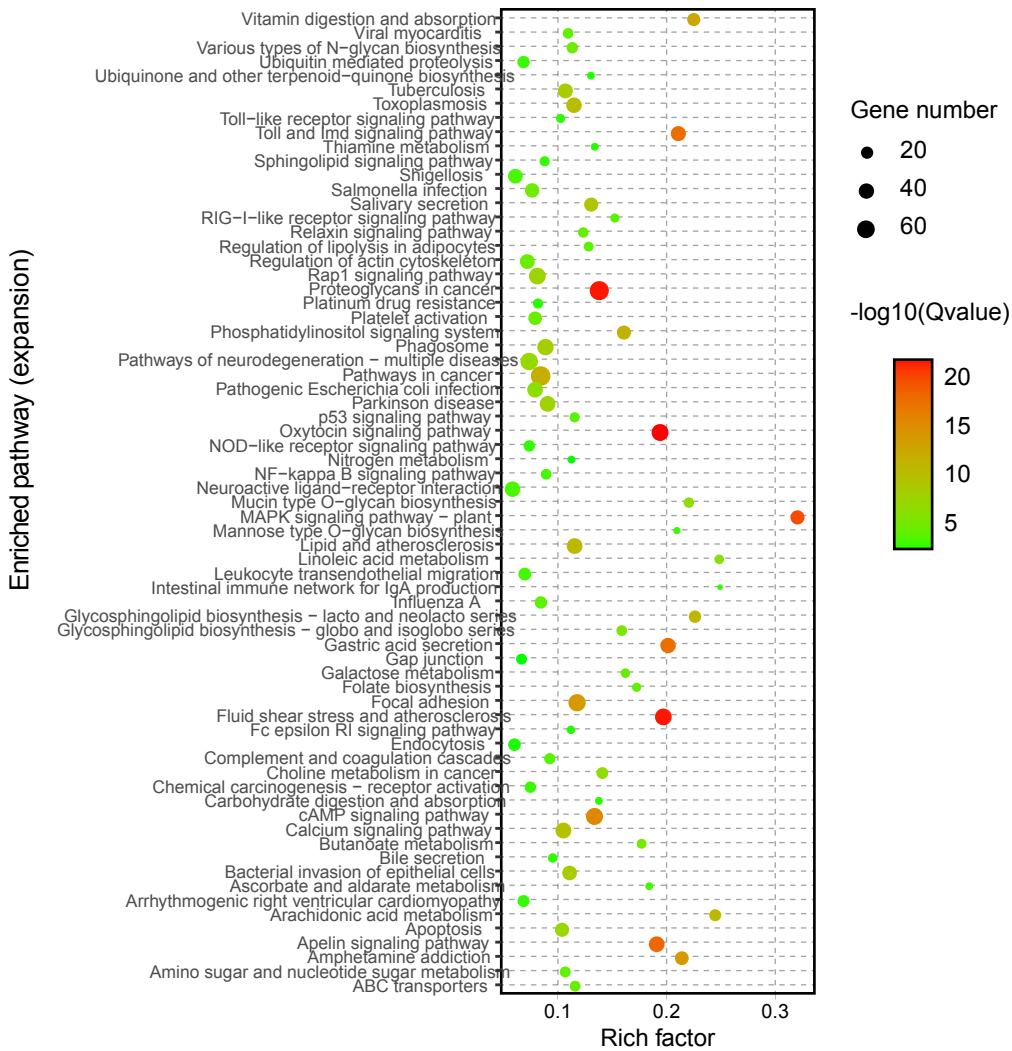

B

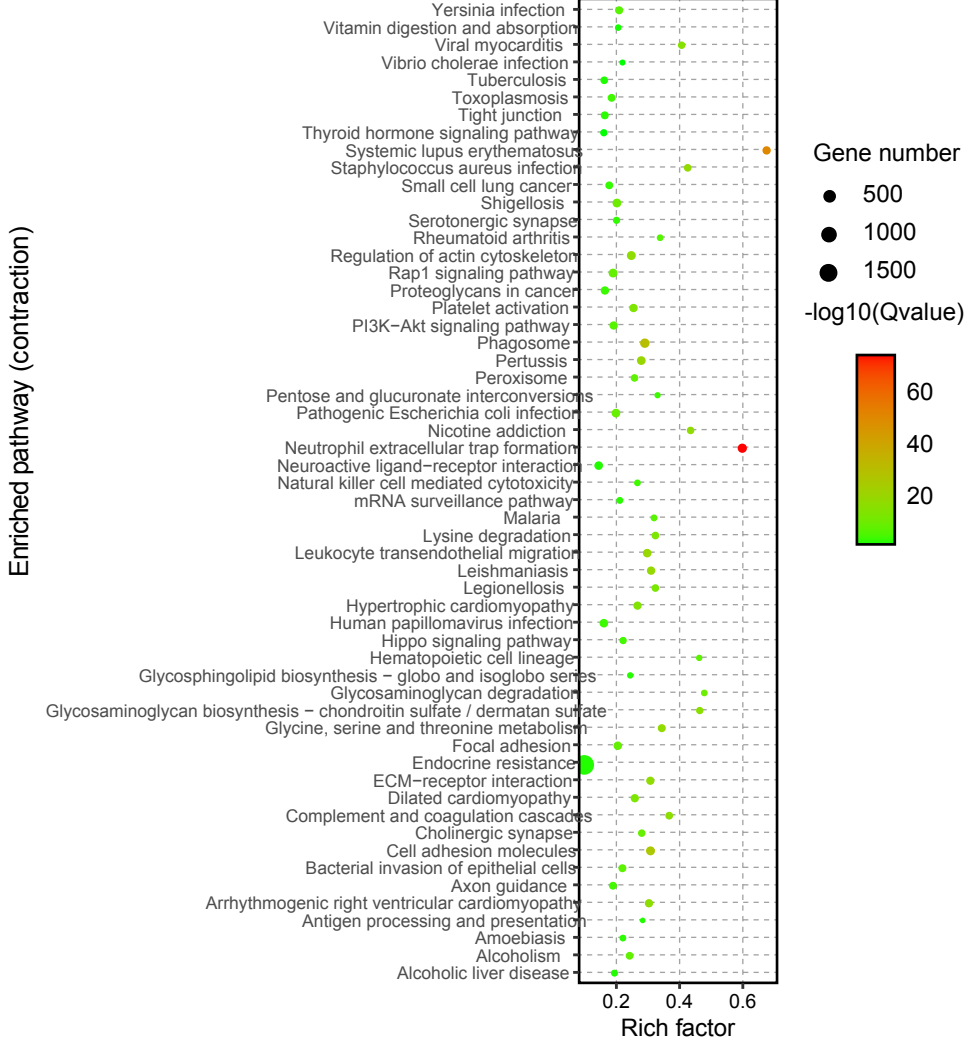

C

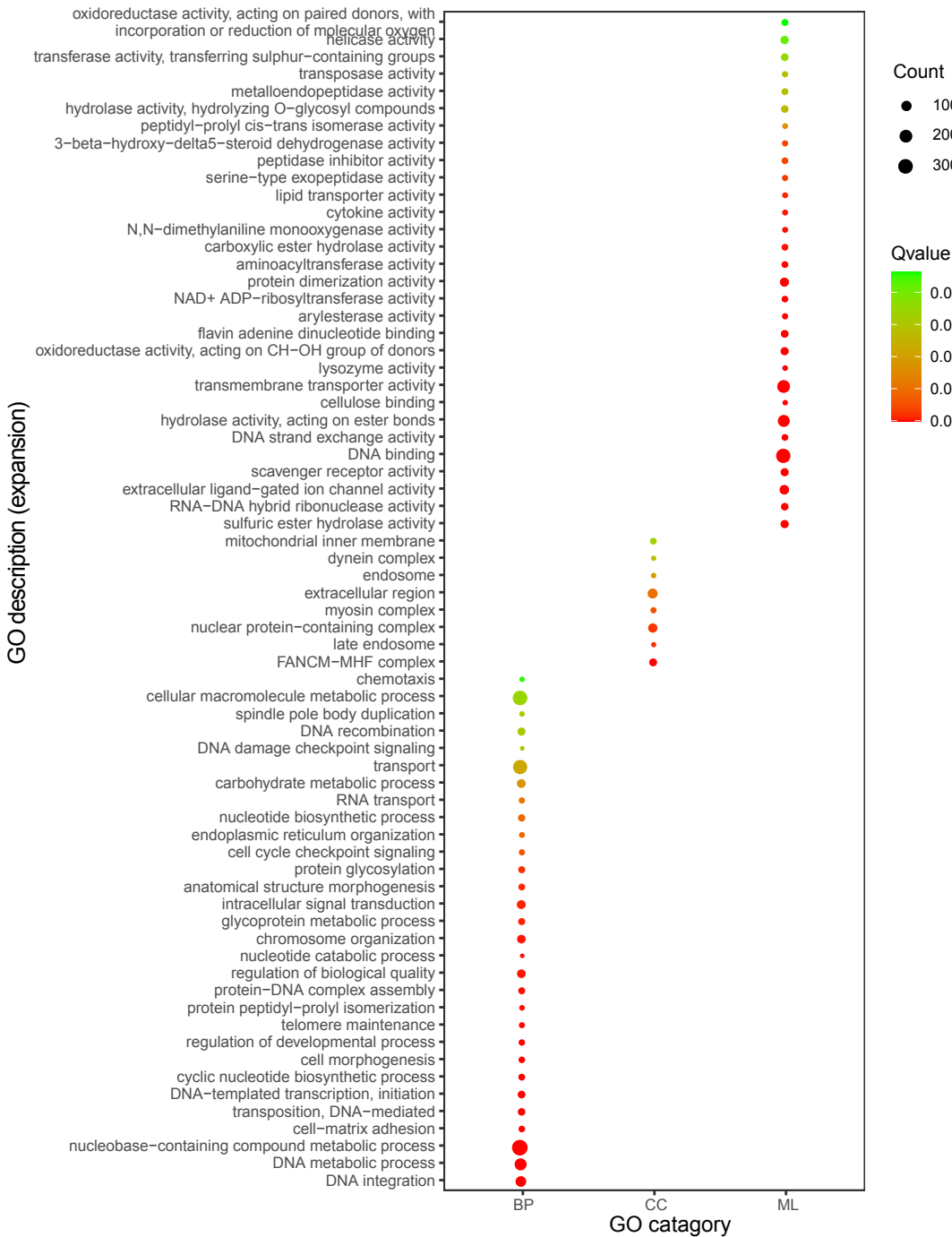

D

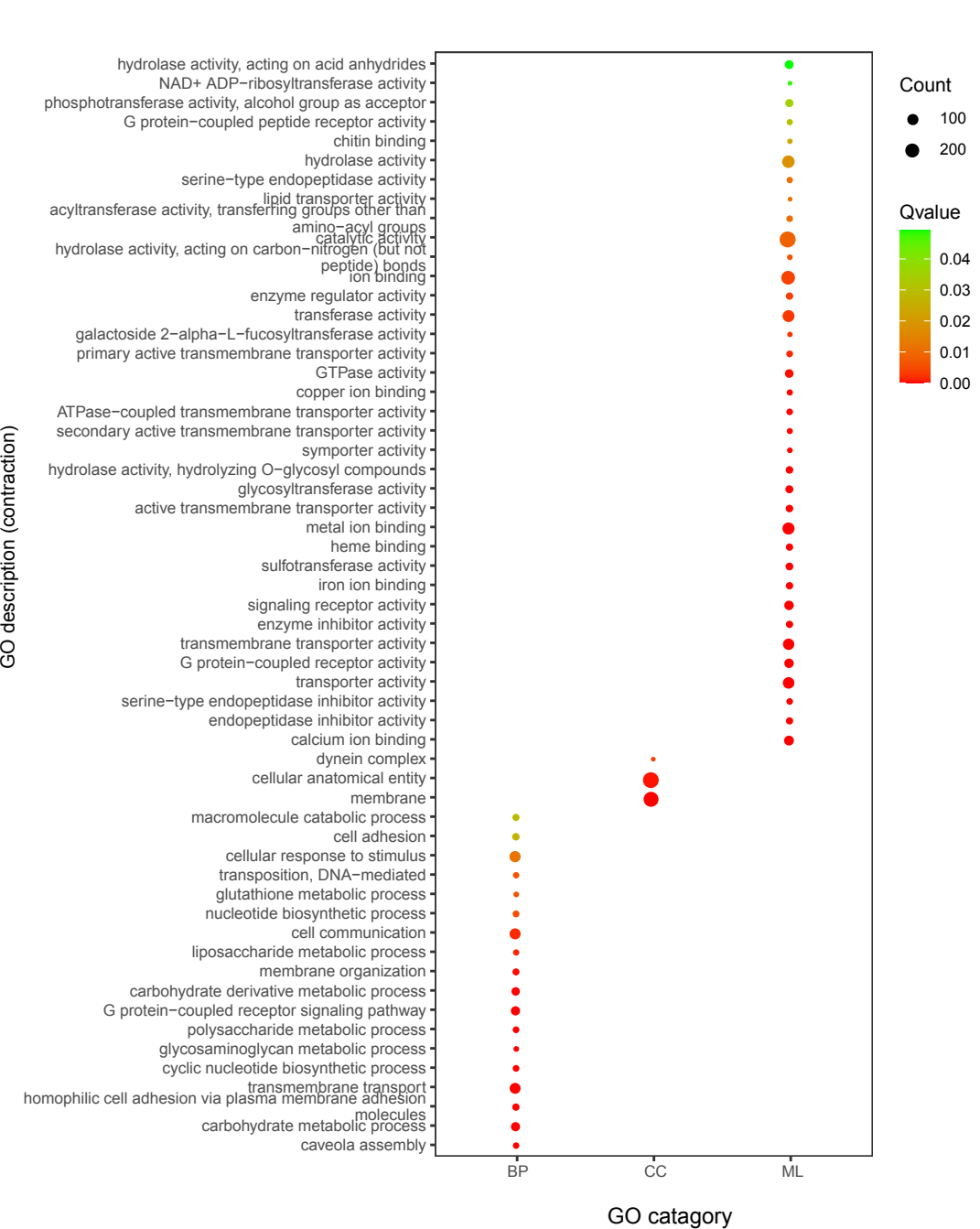

Supplement: jkac229_Supplementary_Figure_S7 [file jkac229_supplementary_figure_s7.pdf]
