# Supplementary material for: A high-quality chromosome-level genome assembly of the bivalve mollusk Mactra veneriformis
Source: G3 (Bethesda). 2022 Sep 27;12(11):jkac229. doi: 10.1093/g3journal/jkac229 (PMC9635629; doi:10.1093/g3journal/jkac229)
Supplement: jkac229_Supplementary_Figure_S5 [file jkac229_supplementary_figure_s5.pdf]

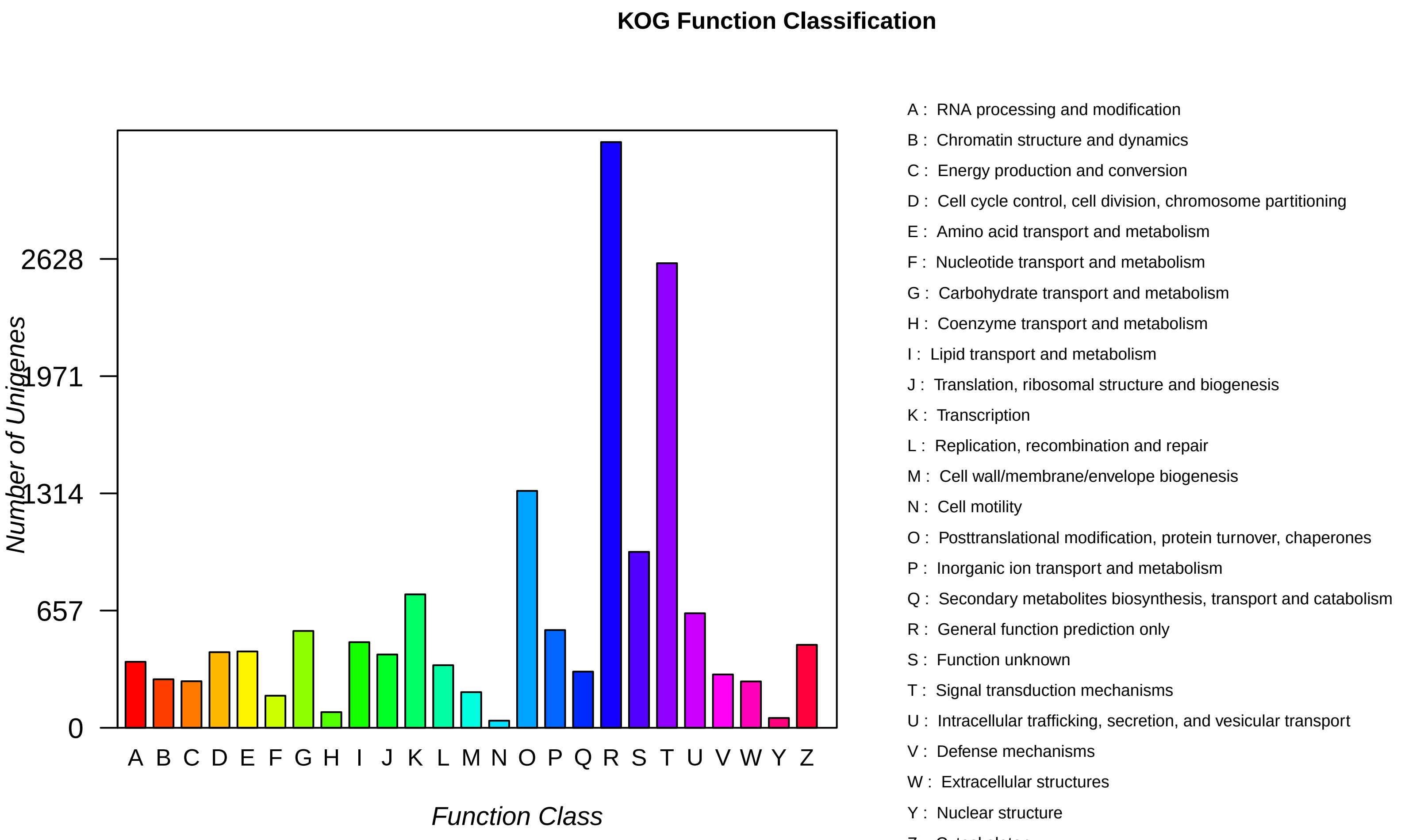
KOG Function Classification
Number of Unigenes
2628
1971
1314
657
0
A B C D E F G H I J K L M N O P Q R S T U V W Y Z
Function Class
A : RNA processing and modification
B : Chromatin structure and dynamics
C : Energy production and conversion
D : Cell cycle control, cell division, chromosome partitioning
E : Amino acid transport and metabolism
F : Nucleotide transport and metabolism
G : Carbohydrate transport and metabolism
H : Coenzyme transport and metabolism
I : Lipid transport and metabolism
J : Translation, ribosomal structure and biogenesis
K : Transcription
L : Replication, recombination and repair
M : Cell wall/membrane/envelope biogenesis
N : Cell motility
O : Posttranslational modification, protein turnover, chaperones
P : Inorganic ion transport and metabolism
Q : Secondary metabolites biosynthesis, transport and catabolism
R : General function prediction only
S : Function unknown
T : Signal transduction mechanisms
U : Intracellular trafficking, secretion, and vesicular transport
V : Defense mechanisms
W : Extracellular structures
Y : Nuclear structure
Z : Cytoskeleton
