# Supplementary material for: A high-quality chromosome-level genome assembly of the bivalve mollusk Mactra veneriformis
Source: G3 (Bethesda). 2022 Sep 27;12(11):jkac229. doi: 10.1093/g3journal/jkac229 (PMC9635629; doi:10.1093/g3journal/jkac229)
Supplement: jkac229_Table_S1 [file jkac229_table_s1.docx]

Table S1. Summary statistics for the clean sequencing data obtained from *M. veneriformis* via Illumina and PacBio sequencing

| Clean Reads | Illumina Sequencing | PacBio Sequencing | HiC Sequencing |
| --- | --- | --- | --- |
| Size of library | 500 bp | 20 kb | 500 kb |
| Number of Reads | 96,217,543 *2 | 37,439,559 | 234,005,013 * 2 |
| Average length of Reads | 147 bp | 11,149 bp | 149 bp |
| Total Bases | 28,308,402,172 bp | 417,402,430,850 bp | 69,676,216,418 bp |
| Sequencing Depth | ~45× | ~400× | ~112× |
