# Supplementary material for: A high-quality chromosome-level genome assembly of the bivalve mollusk Mactra veneriformis
Source: G3 (Bethesda). 2022 Sep 27;12(11):jkac229. doi: 10.1093/g3journal/jkac229 (PMC9635629; doi:10.1093/g3journal/jkac229)
Supplement: jkac229_Table_S2 [file jkac229_table_s2.docx]

| Biological classification | Number of elements* | Length occupied (bp) | Percentage of sequence (%) |
| --- | --- | --- | --- |
| Retroelements | 107,609 | 35,287,674 | 3.60 |
| SINEs: | 1,043 | 101,614 | 0.01 |
| Penelope: | 2,703 | 576,164 | 0.06 |
| LINEs: | 82,517 | 28,803,183 | 2.94 |
| CRE/SLACS | 8 | 393 | 0.00 |
| L2/CR1/Rex | 28,432 | 8,967,491 | 0.92 |
| R1/LOA/Jockey | 11,206 | 6,499,718 | 0.66 |
| R2/R4/NeSL | 1,376 | 176,910 | 0.02 |
| RTE/Bov-B | 30,333 | 9,478,415 | 0.97 |
| L1/CIN4 | 2,490 | 193,654 | 0.02 |
| LTR elements: | 24,049 | 6,382,877 | 0.65 |
| BEL/Pao | 1,208 | 39,244 | 0.04 |
| Ty1/Copia | 4,141 | 267,025 | 0.03 |
| Gypsy/DIRS1 | 14,517 | 5,081,548 | 0.52 |
| Retroviral | 1,795 | 115,428 | 0.01 |
| DNA transposons | 66,523 | 12,409,643 | 1.27 |
| hobo-Activator | 8,266 | 984,782 | 0.10 |
| Tc1-IS630-Pogo | 18,580 | 5,856,995 | 0.60 |
| En-Spm | 0 | 0 | 0.00 |
| MuDR-IS905 | 0 | 0 | 0.00 |
| PiggyBac | 309 | 20,161 | 0.00 |
| Tourist/Harbinger | 3,761 | 463,809 | 0.05 |
| Other(Mirage,  P-element,Transib) | 791 | 34,956 | 0.00 |
| Rolling-circles | 8,591 | 1,301,977 | 0.13 |
| Unclassified | 1,983,252 | 455,012,547 | 46.46 |
| Total interspersed repeats: | | 502,709,864 | 51.33 |
| Small RNA: | 3 | 209 | 0.00 |
| Satellites: | 3,587 | 622,011 | 0.06 |
| Simple repeats: | 13,787 | 2,559,249 | 0.26 |
| Low complexity: | 141 | 26,132 | 0.00 |

Table S2. Summary statistics for the repeat elements found in the *M. veneriformis* genome assembly using both the RepeatModeler and RepeatMasker software
