# Supplementary material for: A high-quality chromosome-level genome assembly of the bivalve mollusk Mactra veneriformis
Source: G3 (Bethesda). 2022 Sep 27;12(11):jkac229. doi: 10.1093/g3journal/jkac229 (PMC9635629; doi:10.1093/g3journal/jkac229)
Supplement: jkac229_Table_S3 [file jkac229_table_s3.docx]

Table S3. Estimation of core genes in the *M. veneriformis* genome assembly using the BUSCO software

|  | Number | Percentage (%) |
| --- | --- | --- |
| Complete BUSCOs | 243 | 95.3 |
| Complete and single-copy BUSCOs | 213 | 83.5 |
| Complete and duplicated BUSCOs | 30 | 11.8 |
| Fragmented BUSCOs | 6 | 2.4 |
| Missing BUSCOs | 6 | 2.3 |
| Total BUSCO groups searched | 255 | 100 |
