# Supplementary material for: A high-quality chromosome-level genome assembly of the bivalve mollusk Mactra veneriformis
Source: G3 (Bethesda). 2022 Sep 27;12(11):jkac229. doi: 10.1093/g3journal/jkac229 (PMC9635629; doi:10.1093/g3journal/jkac229)
Supplement: jkac229_Table_S4 [file jkac229_table_s4.docx]

Table S4. Summary of the orthologous gene clusters analyzed in the 20 different species

| Species name | No. of coding genes | No. of gene families | No. of genes in the gene families | No. of specific gene families | Average genes in the gene families |
| --- | --- | --- | --- | --- | --- |
| *M. veneriformis* | 29,315 | 12,919 | 26,381 | 2,734 | 2.04 |
| *Archivesica marissinica* | 28,949 | 11,308 | 24,852 | 3,955 | 2.2 |
| *Argopecten purpuratus* | 26,256 | 13,922 | 22,969 | 3,268 | 1.65 |
| *Bathymodiolus platifrons* | 33,584 | 12,925 | 29,024 | 4,377 | 2.25 |
| *Chlamys farreri* | 28,602 | 14,142 | 23,776 | 4,807 | 1.68 |
| *Crassostrea gigas* | 30,069 | 11,952 | 24,617 | 4,783 | 2.06 |
| *Cyclina sinensis* | 27,564 | 13,573 | 24,399 | 3,123 | 1.8 |
| *Lottia gigantea* | 23,817 | 10,751 | 16,582 | 6,867 | 1.54 |
| *Mercenaria mercenaria* | 36,095 | 13,856 | 32,419 | 3,560 | 2.34 |
| *Mizuhopecten yessoensis* | 23,930 | 13,662 | 22,339 | 1,586 | 1.64 |
| *Modiolus philippinarum* | 36,549 | 13,350 | 31,978 | 4,405 | 2.4 |
| *Mytilus coruscus* | 58,249 | 14,883 | 47,101 | 10,671 | 3.16 |
| *Mytilus galloprovincialis* | 16,208 | 4,379 | 14,394 | 1,789 | 3.29 |
| *Octopus bimaculoides* | 15,582 | 10,678 | 14,625 | 950 | 1.37 |
| *Octopus sinensis* | 18,088 | 10,380 | 15,125 | 2,765 | 1.46 |
| *Pecten maximus* | 25,165 | 13,317 | 23,054 | 2,097 | 1.73 |
| *Ruditapes philippinarum* | 27,652 | 13,536 | 25,257 | 2,335 | 1.87 |
| *Scapharca broughtonii* | 24,045 | 12,788 | 21,543 | 2,451 | 1.68 |
| *Scapharca kagoshimensis* | 24,908 | 11,895 | 23,770 | 1,113 | 2 |
| *Sinonovacula constricta* | 26,273 | 11,296 | 19,657 | 6,476 | 1.74 |
