# Supplementary material for: A high-quality chromosome-level genome assembly of the bivalve mollusk Mactra veneriformis
Source: G3 (Bethesda). 2022 Sep 27;12(11):jkac229. doi: 10.1093/g3journal/jkac229 (PMC9635629; doi:10.1093/g3journal/jkac229)
Supplement: jkac229_Table_S5 [file jkac229_table_s5.docx]

Table S5. Results of the gene family analysis implemented using the CAFE software

| Species | Expanded families | Significantly expanded  Families (P < 0.05) | Families with no change | Contracted families | Significantly contracted families (*P* < 0 05) |
| --- | --- | --- | --- | --- | --- |
| *M. veneriformis* | 1,501 | 184 | 6,756 | 805 | 219 |
| *Archivesica marissinica* | 1,023 | 146 | 6,388 | 1,627 | 292 |
| *Argopecten purpuratus* | 897 | 157 | 7,603 | 474 | 147 |
| *Bathymodiolus platifrons* | 832 | 173 | 7,314 | 716 | 205 |
| *Chlamys farreri* | 924 | 136 | 7,449 | 582 | 146 |
| *Crassostrea gigas* | 1,030 | 219 | 7,078 | 1,066 | 224 |
| *Cyclina sinensis* | 1,626 | 151 | 6,555 | 809 | 237 |
| *Lottia gigantea* | 1,067 | 130 | 6,823 | 1,564 | 345 |
| *Mercenaria mercenaria* | 1,008 | 296 | 7,087 | 843 | 77 |
| *Mizuhopecten yessoensis* | 661 | 131 | 7,687 | 607 | 147 |
| *Modiolus philippinarum* | 1,644 | 290 | 6,390 | 828 | 115 |
| *Mytilus coruscus* | 1,487 | 375 | 6,503 | 91 | 22 |
| *Mytilus galloprovincialis* | 109 | 49 | 591 | 7,381 | 455 |
| *Octopus bimaculoides* | 678 | 54 | 8,128 | 648 | 173 |
| *Octopus sinensis* | 620 | 96 | 7,917 | 917 | 131 |
| *Pecten.maximus* | 614 | 142 | 7,480 | 880 | 143 |
| *Ruditapes philippinarum* | 1,084 | 171 | 7,052 | 802 | 194 |
| *Scapharca broughtonii* | 571 | 123 | 7,306 | 1,007 | 247 |
| *Scapharca kagoshimensis* | 2,852 | 252 | 5,155 | 877 | 142 |
| *Sinonovacula constricta* | 1,732 | 159 | 5,900 | 1,467 | 267 |
